# Integrating social prescribing in a Canadian regional health system to support healthy aging

**DOI:** 10.24095/hpcdp.44.9.06

**Published:** 2024-09

**Authors:** Margaret Chen-Mei Lin, Grace Park, Maureen C. Ashe

**Affiliations:** 1 Fraser Health, Surrey, British Columbia, Canada; 2 Department of Family Practice, The University of British Columbia, Vancouver, British Columbia, Canada

**Highlights**

- British Columbia (BC) has developed a province-wide social prescribing model supporting older adults through close partnerships between health care and community organizations.
- A regional health authority, Fraser Health, has a specific regional team focussing on integrating social prescribing into the health system through meaningful engagement and continuous co-creation with multisectoral partners, using strategies such as change management and Plan-Do-Study-Act cycles.
- Environmental and organizational support are big facilitators that have supported the continuation of the designated integration effort.
- Long-term funding and more partnerships between health care and community organizations will be critical to sustaining the social prescribing model in BC.

## Introduction

Social prescribing (SP) is a rapidly growing health and social model of care. The concept of social prescribing is based on well-known clinical practices such as community referrals, integrated health and social care,^1^ and navigator models.^2^,^3^ Although SP began in the United Kingdom’s mental health and social care field, there are many examples of different models of SP foci and pathways.^4^ Here in Canada, SP is emerging at several provincial locations, with differences in its delivery reflecting the local context of people and places. 

In British Columbia (BC), there are five regional health authorities, a First Nations health authority and a provincial health authority overseeing specialized services. Fraser Health is the largest of the five regional health authorities in BC. It employs 45 000 health workers, delivering hospital- and community-based health services to more than 1.9 million people in 20 diverse communities, including over 320 000 adults over 65 years of age.^5^ Since 2019, Fraser Health has been partnering with United Way BC (UWBC) to support the integration of SP into practice for older adults. 

We provide a summary of the experience of the Fraser Health team, who are integrating SP into practice, to inform other health organizations, policy makers, decision-makers and health care providers who may be beginning a similar process. We describe the development and structure of the BC social prescribing model, followed by a summary of the team’s strategies to support SP model integration. We conclude this work with reflections on the strengths and challenges we encountered during the regional integration work.

## Co-creating social prescribing in BC and the Fraser Health region

The SP model at Fraser Health was developed through a partnership with the provincial government, UWBC, other nonprofit community organizations, BC Divisions of Family Practices (representing primary care physicians in the province) and Fraser Health teams including the Patient and Family Advisory Council. The partnership began in 2019, when the BC Ministry of Health provided funding for a new program through UWBC.^6^ At the time, Fraser Health had begun an initiative called “Community Actions and Resources Empowering Seniors” (CARES), which engages community-based primary care providers to identify, manage and develop care plans for older adults living with frailty, based on evidence that it can be delayed or prevented.^7^ The initiative piloted the model of care in two local communities to spread the innovation throughout the region. A partnership between CARES and UWBC allowed Fraser Health community practitioners to further partner with local nonprofit organizations to create a new peer support role to help older adults navigate available services, called the “seniors community connector” (SCC). 

Between 2019 and December 2023, there were 20 SCCs working throughout BC. The SCCs are staff hired by local community nonprofit organizations, although their position is funded by a BC Ministry of Health grant and managed through UWBC.^6^ The SCCs share many common features with the community link workers in the UK SP model.8 For example, they use a strengths-based approach to address unmet, nonmedical social needs; locate and connect older adults with community resources; and follow up over time.^8^ The SCCs come from a variety of backgrounds and have a variety of training, such as social work and nursing, or experience in the nonprofit sector. 

The SCCs from each catchment area receive referrals from health care providers, older people or their friends and families. Based on the older adult’s needs and preferences, the SCC provides tailored support to facilitate access to community resources, which may range from food and nutrition support to physical activity and social engagement opportunities in the older adult’s community. After the initial meeting, the older person and SCC set goals, co-create a wellness plan, and develop a follow-up plan together.

## Integrating social prescribing into the health system 

***The role of the Fraser Health social prescribing team***

At Fraser Health, a SP team evolved from the CARES initiative to support the integration of social prescribing into the regional health system. The Fraser Health SP team includes eight core team members who meet weekly to monitor project progress and discuss strategies. Two SP “change leads” are employed by Fraser Health to fully support the initiative. The aim of this role is to implement and facilitate organizational improvements through change management strategies. One lead has experience as a clinical nurse educator in community health and the other lead is a registered nurse who coordinated the CARES initiative. The two change leads are supported by six team members with already existing roles in Fraser Health’s Home and Community Services regional team: a service operations director, a regional medical director, a clinical nurse specialist, a clinical nurse educator, a clinical social work educator and an occupational therapy clinical leader. The team also works with consultants from the Communications and System Optimization department within Fraser Health.

The SP team is critical to the integration of a complex model like social prescribing, as the team members act as implementation support practitioners (ISPs), which have been shown to be beneficial by implementation science studies.^9^-^11^ An ISP is a “facilitator, coach, knowledge broker and technical assistance provider to support implementation of evidence-informed programs and practices … to sustain and scale research evidence for improved and equitable population outcomes.”^11^^,p.2^ At Fraser Health, the two change leads take on the role of ISP and co-create strategies with partners to integrate SP, apply ongoing quality improvement and support the sustainability of the program.^12^

Integrating new programs into practice involves multiple phases, such as the ISPs providing information on the program (knowledge mobilization) to service providers, and the providers adopting and sustaining the new intervention.^13^ The SP team facilitates these phases and engages partners using the ISP core competencies derived from implementation science and quality improvement strategies, such as the Plan-Do-Study-Act (PDSA) cycle from improvement science.^14^ Utilizing both types of strategies promotes engagement, fosters local ownership and helps refine strategies.^15^

***Engagement process***

**Figure 1:**
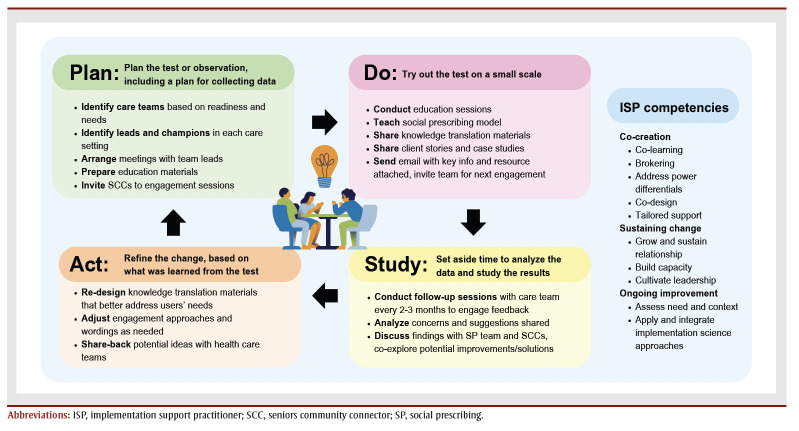
Fraser Health social prescribing team engagement process

The SP team’s process of health care staff engagement using PDSA cycles and ISP competencies is summarized in Figure 1. Along with frontline care providers, the team engages other regional team members in Fraser Health to consider how the SP model can complement existing services in primary care, community health, public health and palliative care settings to support older adults’ quality of life. To ensure a suitable and sustainable SP model in Fraser Health, the change leads engage SCCs regularly through monthly meetings that form communities of practice that aim to share insights and resources from the health care sector with the SCCs, hear the SCCs’ insights, encourage peer support among the SCCs and co-create action plans to improve the health care–community partnerships. The SP team also partners with other nonprofit community organizations, universities, members of the public and national SP organizations, such as the Canadian Institute of Social Prescribing, to explore collaborations. For instance, some local BC universities have started to embed SP as a topic in health care students’ curricula and encourage preceptorships in SP. 

All materials shared and knowledge disseminated continue to be developed based on users’ feedback (health care providers and SCCs) throughout the engagement process. The change leads facilitate communication between health care teams and the SCCs, provide tailored support and suggest ways for the two sectors to collaborate. For example, early in the engagement process, the Fraser Health SP team quickly realized that the health care system and community service organizations have different workflow and infrastructure systems. Health care providers often identify and request a specific type of community resource for patients and prefer a standardized referral process in which each step of the program is clearly defined. On the other hand, the SP model encourages holistic exploring of nonmedical needs and approaches, and the time and approach required to do this varies highly based on the individuals’ needs and community resources. Brown et al.^16^ also noted that “[t]he formalization of social prescribing within [the health system] … and the administrative activities that this is likely to bring with it … could endanger some of the existing advantages of [community work], such as its flexibility, informality and personal approach.”^16^^,p.621^

Upon realizing the difference, the Fraser Health SP team quickly developed standardized ways to clarify the nature and scope of SP and took time to explain to both the health care and the community care providers the difference in expectations for components such as referral criteria, program scope and follow-up mechanisms, and offered recommendations to facilitate collaboration. 

## Reflections

In addition to engaging partners, the SP team constantly reflects on the integration journey and takes action to develop a more sustainable SP model in BC and Fraser Health. Between September 2019 and July 2023, the SP program in the Fraser Health region was introduced to at least 126 health care teams, and supported over 1000 older adults in the region. We are aware that for SP to support more people, sustainability and maturity of the model are critical. In this section, we reflect on our experience and learnings based on the eight domains suggested by the Program Sustainability Assessment Tool (PSAT),^17^ which evaluates and aids sustainability planning for public health programs: environmental support, funding stability, partnerships, organizational capacity, program evaluation, program adaption, communications and strategic planning. 

***1. Environmental support***

Like the rest of Canada, BC and the Fraser Health region have an aging population.^18^,^19^ The BC Ministry of Health and other interested parties in the health care industry have been exploring different ways to support interdisciplinary care and healthy aging. Within Fraser Health, there has also been an emphasis on enhancing the integration of a community services model with the traditional model focussed on acute care.^20^ This health care trend and supportive environment have led to the integration and promotion of the SP model in the Fraser Health community. When we introduced the SP model to health care and community staff, leadership teams and the public in the past, we received positive feedback and enthusiasm, showing that this model is timely and aligns with people’s needs. In the next phase, we will engage with municipalities to further explore (and hopefully integrate) the health–community partnership model.

***2. Funding stability***

Since the start of the program, the SP program has been funded as a demonstration project on a year-to-year basis by both the Ministry of Health and Fraser Health. The uncertainty of continuous funding had led to concerns from care providers in the community and within Fraser Health. The SP team continues to advocate for continuous funding, by highlighting program gaps in reports and in meetings with both health care and community decision makers. At the time of writing, the Ministry of Health and Fraser Health had released new funding to support the initiative, and the UWBC and Fraser Health team members are continuously brainstorming on how to optimally streamline health care and community infrastructures. 

***3. Partnerships***

Partnerships with different health care teams and community organizations have been one of the biggest levers in SP integration. Partners have been supportive, leading to more opportunities to collaborate on a healthy aging environment, including leveraging existing resources. A barrier the SP team encountered is that SCCs in the community and health care providers do not have a mutual platform or standardized method and policy for information sharing, leading to difficulty in collaborative care planning and follow-up. The SP team continues to explore different ways to facilitate communication between health care staff and community organization staff. 

***4. Organizational capacity***

Support from Fraser Health has been critical to sustaining the integrated SP model. The ease of accessing and partnering with other Fraser Health teams has led to better integration of services. For example, the team has support from the Fraser Health communications department to facilitate knowledge mobilization. The team also partners with the health authority’s systems optimization and research teams for program evaluation.

***5. Program evaluation***

With support from fellow Fraser Health team members, the SP team is evaluating the program’s impact on older adult health and health system utilization, such as emergency room utilization and hospital re-admission rate. We continue to partner with UWBC, local community organizations and Fraser Health team members to evaluate program capacity and sustainability. We also plan on evaluating the experience of older adults, family and care providers with social prescribing, via surveys.

***6. Program adaption***

The SP initiative in Fraser Health adapts rapidly based on feedback, new partnerships and health system needs. The SP team values and respects the readiness and capacity of each interested party and adjusts integration approaches through continuous engagement and PDSA cycles. Our learnings also lead to discussion about whether more health authority staff should be hired or trained to specifically address more urgent nonmedical needs during care transition, in addition to the existing SP model. We aim to continue adapting the SP program based on routinely collected data, creating a “learning health system” in social prescribing.

***7. Communications***

Standardized messages and promotional materials greatly facilitate learning and adaption of the health care–community model. The SP team is partnering with the Fraser Health communications department and UWBC to build standardized materials for the SCCs, health care providers, and older adults and families. We have used methods such as social media campaigns, community sessions and conferences to increase community awareness, and hope to have more opportunities to showcase the social–health model on a larger scale. 

***8. Strategic planning***

The Fraser Health 2020/21–2022/23 Service Plan highlighted the Authority’s priority of ensuring older adults access to timely and comprehensive care through increased partnership between community and health care.^20^ This priority prompted a new initiative, Frailty Pathway, which includes the SP model and related collaborative initiatives to address frailty in Fraser Health. The initiative is leading to increased funding from the health authority and the formation of new partnerships in order to establish a comprehensive healthy aging care model that supports more older adults. 

We are grateful for the collaboration of our partners, which allowed us to develop social prescribing in Fraser Health. Continued engagement with health care, community organization and academic institution partners and the willingness of all partners to co-create strategies have been the key element leading to our success to date. Although each system and community structure is unique, we believe our learnings and practical, evidence-informed strategies will inspire other health systems to embed social prescribing in their region.

## Acknowledgements

We would like to thank the 19 community organizations and 20 seniors community connectors who are delivering social prescribing in BC, and the Province of British Columbia and United Way BC for the funding and coordination of the social prescribing programs in BC. We would like to thank all of the Fraser Health team members for their ongoing support and partnerships to integrate social prescribing together in Fraser Health. MCA gratefully acknowledges the support of the Canada Research Chairs Program.

## Conflicts of interest

ML is the social prescribing change lead and Frailty Pathway project lead employed by Fraser Health. GP is the regional medical director for Home and Community Services, contracted by Fraser Heath. MCA is a professor at The University of British Columbia, with no conflicts to declare.

## Authors’ contributions and statement

ML, GP, MCA: conceptualization, formal analysis, methodology, visualization, writing—original draft, writing—review and editing.

ML, GP: project administration, resources.

The content and views expressed in this article are those of the authors and do not necessarily reflect those of the Government of Canada.
